# Supplementary material for: Prevalence of Congenital Disorders of Glycosylation in Childhood Epilepsy and Effects of Anti-Epileptic Drugs on the Transferrin Isoelectric Focusing Test
Source: Genes (Basel). 2021 Aug 10;12(8):1227. doi: 10.3390/genes12081227 (PMC8391492; doi:10.3390/genes12081227)
Supplement: Supplementary file 1 [file genes-12-01227-s001.zip › genes-1234092-supplementary.pdf]

Table S1: Clinical features, neuroimaging, and genetic test results of all patients with genetically confirmed childhood epilepsy identified in this study are summarized.

**Number/study ID/diagnosis/sex/age/consanguinity**	**Presenting symptom (age of onset)/other clinical features**	**EEG**	**Neuroimaging**	**Molecular genetic test result (number of genes in the TNGSP)**
1/001/*STXBP1* disease/F/7yrs/no	Seizures (GTCS, GTS, GCS, focal) (18mo), GDD, ASD, aggressive behavior	SpW	Ventriculomegaly	De novo c.560C>T (p.Pro187Leu) in *STXBP1* by TNGS (127)
2/004/*WDR45* disease/F/13yrs/no	GDD (15 mo), seizure (GTCS, focal) (2 yrs), MD (dystonia, spasticity)	BG asymmetry	Thin CC, cerebellar atrophy, low NAA	De novo c.400C>T (p.Arg134*) in *WDR45* by ES
3/009/*GLUT1* disease/M/13yrs/no	Seizures (AbS, MS, AS, GTS) (12mo), GDD, MD (ataxia), ASD, ADHD	GSpW, PSpW, FIRDA	Increased T2 signal in subcortical WM	De novo c.940G>A (p.Gly314Ser) in *SLC2A1* by ES
4/012/*GLUT1* disease/M/4yrs/no	Seizures (GTCS) (5mo), GDD	SBG	N	De novo c.656delA (p.Asn219Thrfs*10) in *SLC2A1* by ES
5/013/*CACNA1A* disease/F/10yrs/no	Seizures (AbS, AS) (2mo), GDD	SpW, SW	N	De novo c.4253G>A (p.Arg1418Gln) in *CACNA1A* by ES

6/015/*SLC35A2*-CDG/F/2yrs/no	FTT (day 3), GDD, Seizures (IS) (11mo), dysmorphic features (hypertelorism, low set posteriorly rotated ears, prominent forehead, upslanting palpebral fissures, short nose with upturned nose tip)	Modified HypA, SBG, GSp, MFSp, SW	DM, hypoplastic BS	De novo c.3G>A (p.Met1?) in *SLC35A2* by ES
7/018/*DLAT* disease/M/9yrs/no	Seizures (GTS, MS, GCS, AbS) (2mo), GDD, MD (dystonia, spasticity)	SW, SBG	Microcephaly, DM, frontal lobe atrophy	Unknown c.1689delT (p.Phe563Leufs) in *DLAT* by TNGS (18)
8/046/*ATP1A2* disease/M/6yrs/no	Seizures (GTCS) (18mo), GDD	N	Chiari I malformation, Increased T2 signal in frontal WM, parietal WM, cerebellum	Maternal (mother affected) c.1091C>T (p.Thr364Met) in *ATP1A2* by TNGS (87)
9/060/*NEXMIF* disease/F/11yrs/no	Seizures (AbS, MS) (14mo), ADHD	GSSW	N	Unknown c.2163delA (p.Lys721Asnfs*8) in *NEXMIF* by TNGS (127)
10/061/*SYNGAP1* disease/F/10yrs/	Speech delay (18mo), GDD, Seizures (AbS) (2yrs)	GSpW	N	De novo c.1490A>G (p.Tyr497Cys) in *SYNGAP1* by TNGS (127)

11/067/*KCNQ2* disease/M/9yrs/no	Seizures (GTCS, GTS) (day 1), GDD, MD (dystonia, spasticity)	SW, SBG	N	Not maternal, paternal NA c.821C>T (p.Thr274Met) in *KCNQ2* by ES
12/070/*DDX3X* disease/F/17yrs/yes	GDD (18mo), Seizures (GTCS) (13yrs), MD (motor apraxia), ASD	GPSpW	Increased T2 signal in right insular, dysgenetic CC, cerebellar volume loss	De novo c.641_643delinsCC (p.Ile214Thrfs*7) in *DDX3X* by ES
13/085/SCN1A disease/M/3yrs/no	Seizures (GTCS, GTC, focal) (4mo), GDD	BG asymmetry, SW	N	De novo c.635T>C (p.Val212Ala) in *SCN1A* by TNGS (127)
14/091/18q deletion syndrome/M/9yrs/no	Seizures (focal) (22mo), GDD, dysmorphic features (flat nasal bridge, prominent jaw, small posteriorly rotated ears, hypertelorism)	SpW, SW	DM	18q22.1q23 deletion by microarray
15/095/*NEXMIF* disease/F/14yrs/no	GDD (6mo), Seizures (AS, AbS) (5yrs), ASD, ADHD, OCD, separation anxiety	SBG, SpW	N	De novo c.114C>T (p.Arg481*) in *NEXMIF* by ES
16/104/*FOXG1* disease/M/3yrs/no	Seizures (GTS, IS) (2.5mo), GDD	SBG	Microcephaly, dysmorphic CC, thinned hippocampus	Not maternal, paternal NA c.586C>T (p.Gln196*) in *FOXG1* by TNGS (127)

17/109/*CNKSR2* disease/M/9yrs/no	Seizures (GTCS) (3.5yrs), GDD, ADHD	Sp, SW	NP	Maternal c.114delG (p.Ile39Serfs*14) in *CNKSR2* by ES
18/117/Angelman syndrome (*UBE3A* disease)/M/7yrs/no	Seizures (GTS, AS) (day 7), GDD, microcephaly, prognathia	GSpW, PSpW	Thinned CC	De novo c.1396A>G (p.Lys466Glu) in *UBE3A* by TNGS (87)
19/131/*KCNQ2* disease/F/11yrs/no	Seizures (GTS) (day2), GDD, MD (ataxia), ASD	Prolonged attenuations of cortical activity	N	De novo c.700A>C (p.Thr234Pro) in *KCNQ2* by DS
20/132/*KMT2B* disease/M/17yrs/no	GDD (12mo), Seizures (atypical) (6yrs), MD (dystonia, dyskinesia), ASD, self-harm, aggressive behaviour	SpSW	Increased FLAIR signal in PV WM	De novo c.7551-14_7561del25 in *KMT2B* by ES
21/139/*KCNB1* disease/M/12yrs/no	GDD (6mo), Seizures (GTCS, IS, MS, AS, focal, reflex) (12mo), GDD	SBG, Sp, SPSW	Increased T2 signal in left frontal and peritrigonal WM	De novo c.1222C>G (p.Pro408Ala) in *KCNB1* by ES
22/142/*PPP2R5D* disease/F/6yrs/no	Prematurity with fetal tachycardia, Seizures (MS) (day 3), GDD	Discontinuous BG, SW	PV WM atrophy, thinned CC, increase T2 signal in right thalamus	De novo c.592G>A (p.Glu198Lys) in *PPP2R5D* by ES
23/155/*TCF20* disease/F/5yrs/no	Seizure (MS, GTS) (2yrs), GDD, MD (intention tremor)	GPSpSW, SpW, PSpW	Mild cerebellar atrophy	De novo c.3997delC (p.Leu1333Serfs*18) in *TCF20* by ES

24/157/*SCN1A* disease/F/8yrs/no	Seizures (GTCS, AbS, MS, focal) (2mo), GDD, ASD	SpSW, SBG	N	Unknown c.4934G>A (p.Arg1645Gln) in *SCN1A* by TNGS (3)
25/158/*NEXMIF* disease/F/19yrs/no	Unknown, Seizures (GTCS, dyscognitive) (11yrs), GDD, anxiety disorder, self-harm, aggressive behaviour	Sp, PSp	Increased T2 signal in left hippocampus	Unknown c.336G>A (p.Trp112*) in *NEXMIF* by ES
26/159/*GRIN2B* disease/M/22yrs/no	Seizures (GTCS, AS, AbS) (2 yrs), GDD, MD (tic disorder), ADHD, ASD, BPD, aggressive behaviour	N	Thinned CC	Unknown c.3883C>T (p.Arg1295Trp) in *GRIN2B* by TNGS (87)
27/166/*GABRA5* disease/M/5yrs/no	Seizures (MS, focal) (3mo), GDD, MD (choreoathetosis)	PSpW	Microcephaly, thinned CC, Increased T2 signal in frontal and parietal WM	De novo mosaic c.902C>G (p.Thr301Arg) in *GABRA5* by ES
28/177/*SLC6A1* disease/F/4yrs/no	GDD (3mo), Seizures (AS, MS) (9mo), ASD	Sp	N	De novo c.881_883delTCT (p.Phe294del) in *SLC6A1* by TNGS (127)
29/182/*MECP2* disease/F/11yrs/yes	GDD (6mo), Seizures (GTCS) (2yrs), MD (dystonia)	GSSW, SBG, Sp, SW	Microcephaly, reduced NAA	De novo c.763C>T (p.Arg255*) in *MECP2* by ES

30/193/*HIVEP2* disease/F/11yrs/no	GDD (6mo), Seizures (GTCS, AbS, MS) (20mo), ADHD	SpW	N	De novo c.6871C>T (p.Gln2291*) in *HIVEP2* by ES
31/194/*RNASEH2C* disease/F/6yrs/no	Microcephaly (day 1), GDD, seizures (GTCS) (5mo), MD (dystonia, spasticity CP)	SBG, Sp	Microcephaly, thinned CC, calcification of basal ganglia	Cmp Htz<br>Maternal c.205C>T (p.Arg69Trp); paternal c.348+5G>A in *RNASEH2C* by TNGS (7)
32/200/*MBOAT7* disease/F/7yrs/no	Seizures (AS, GTCS, MS) (2mo), GDD, aggressive behaviour	MISF	Increased T2 signal in GP	Cmp Htz<br>Maternal c.758_778del21 (p.Glu253_Ala259del); Paternal c.680_690dup11 (p.Leu231Cysfs*8) in *MBOAT7* by ES
33/202/*SCN2A* disease/F/2yrs/no	Seizures (GTS) (3mo)	SBG, SW	N	Paternal c.2659G>A (p.Val887Ile) in *SCN2A* by TNGS (127)
34/205/*SCN2A* disease/F/4yrs/no	Seizures (GTCS) (3mo), GDD	SBG	Thinned CC, DM	Paternal (father affected) c.2828_2829delGGinsAT (p.Trp943Tyr) in *SCN2A* by TNGS (87)

35/211/*PMM2*-CDG/M/18yrs/no	GDD (3mo), Seizures (GTCS, MS, CPS) (3.5yrs), MD (ataxia), scoliosis, pectus carniatum, kyphosis	N	Cerebellar atrophy, brainstem atrophy, small pons	Cmp Htz c.710C>T (p.Thr237Met); c.447+5G>A (variant IVS5+5G>A) in *PMM2* by ES
36/230/*PMM2*-CDG/M/3yrs/no	FTT (6 wks), GDD, Seizures (GTCS) (18mo), MD (spasticity), dysmorphic features (inverted nipples, low-set ears, asymmetric chest wall)	NP	Thinned CC, cerebellar atrophy, small pons	Cmp Htz c.422G>A (p.Arg141His); c.43G>A (p.Gly15Arg) in *PMM2* by DS

Abbreviations (listed alphabetically):

AbS= absence seizures; ADHD= attention-deficit/hyperactivity disorder; AS = atonic seizures; ASD= autism spectrum disorder; BG= background; BS= brainstem; CC= corpus callosum; CDG= congenital disorders of glycosylation; Cmp=compound; CP= cerebral palsy; CPS= complex partial seizures; DM= delayed myelination; EEG= electroencephalography; F= female; FIRDA= frontal intermittent rhythmic delta activity; FTT= failure to thrive; GDD= global developmental delay; GP= globus pallidus; GSp= generalized spikes; GPSpSW= generalized polyspike-and-slow waves; GSpW= generalized spike-and-waves; GSSW= generalized spike-and-slow-waves; GTCS= generalized tonic-clonic seizures; GTS= generalized tonic seizure; Htz= heterozygous; HypA= hhypsarrhythmia; IS= infantile spasms; mo= months; M= male; MD= movement disorder; MFSp= multifocal spikes; MISF= multiple independent spike foci; MRI= magnetic resonance imaging; MS= myoclonic seizure; N= normal; N/A= not available; NAA= N-acetylaspartic acid; NP= not performed; PSpW = polyspike-and-waves; PV= periventricular; SBG= slowing of background; Sp=

spikes; SpSW= spike-and-slow waves; SpW= spike and waves; SW= sharp waves; TNGS = targeted next-generation sequencing panels; ES= exome sequencing; wk= weeks; WM= white matter; yrs= years

Table S2. Please refer to our previously published studies for the results of the 48 patients with genetic diagnoses, who were included in the current study.

**Study ID(s)**	**References**
002, 014, 017, 020, 025, 032, 033, 041, 056, 057, 058, 073, 081, 082, 083, 090, 102, 106, 111, 112, 118, 119, 120, 122, 130, 138, 144, 145, 146, 148, 150, 151, 172, 173, 174, 181,184, 190, 191, 195, 197, 203	Costain 2019
031, 210, 215, 222, 224	Al Teneiji 2017
165	Jilani 2019

References

Al Teneiji, A. et al. Phenotypic and genotypic spectrum of congenital disorders of glycosylation type I and type II. *Mol Genet Metab* **120**, 235-242 (2017).

Costain, G., Cordeiro, D., Matviychuk, D. & Mercimek-Andrews. S. Clinical Application of Targeted Next-Generation Sequencing Panels and Whole Exome Sequencing in Childhood Epilepsy. *Neuroscience* **418,** 291-310 (2019)

Jilani, A. et al. High diagnostic yield of direct Sanger sequencing in the diagnosis of neuronal ceroid lipofuscinoses. JIMD Rep. 50, 20-30 (2019).

Table S3: *In silico* analysis of variants in genes identified in patients with childhood epilepsy are listed in Table S3.

**Gene NM# (reference)**	**Study ID/variant**	**SIFT**	**MutTaster**	**PolyPhen-2 (HumVar)**	**Conservation in species (Amino Acid)**	**gmAD allele count in allele number**	**Variant Classification**
ALG3 (no NM# in the result)(1)	215/maternal and paternal (homozygous) c.165C>T (p.Val54fs*66)	NA	NA	NA	NA	0	Pathogenic (PVS1, PS3, PM2)
ALG11 (NM _001004127.2)(1)	031/maternal and paternal (homozygous) c. 1241T>A (p.Ile414Asn)	Damaging	Disease-Causing	Possibly Damaging	9 out of 10	0	VUS (PM2, PP3, PP2)
ATP1A2 (NM_000702.3)	046/maternal (symptomatic) c.1091C>T (p.Thr364Met)	Damaging	Disease-Causing	Probably Damaging	7 out of 7	0	Likely Pathogenic (PS2, PM2, PP2, PP3, PP5)

CACNA1A (NM_001127221.1)	013/de novo c.4253G>A (p.Arg1418Gln)	Tolerated	Disease-Causing	Benign	5 out of 7	0	VUS (PM2, PM6)
CNKSR2 (NM_014927.3)	109/maternal (hemizygous) c.114delG (p.Ile39Serfs*14)	NA	NA	NA	NA	0	Pathogenic (PVS1, PM2, PP5)
DDX3X (NM_001356.4)	070/de novo c.641_643delinsCC (p.Ile214Thrfs*7)	NA	NA	NA	NA	0	Pathogenic (PVS1, PM2, PM6, PP5)
DLAT (did not have NM on test)	018/unknown c.1689delT (p.Phe563Leufs)	NA	NA	NA	NA	0	Likely Pathogenic (PVS1, PM2)
FOXG1 (NM_005249.3)	104/unknown c.586C>T (p.Gln196*)	NA	NA	NA	NA	0	Pathogenic (PVS1, PM2, PP5)
GABRA5 (NM_000810.3)	166/de novo mosaic c.902C>G (p.Thr301Arg)	Damaging	Disease-Causing	Probably Damaging	8 our of 9	0	Likely Pathogenic (PM2, PM6, PP2, PP3)

GRIN2B* *(NM_000834.3)	159/unknown c.3883C>T (p.Arg1295Trp)	Damaging	Disease-Causing	Probably Damaging	8 out of 9	1 in 251466	VUS (PM2, PP3)
HIVEP2* *(NM_006734)	193/de novo c.6871C>T (p.Gln2291*)	NA	NA	NA	NA	0	Pathogenic (PVS1, PM2, PM6)
KCNB1* *(NM_004975.2)	139/de novo c.1222C>G (p.Pro408Ala)	Damaging	Disease-Causing	Probably Damaging	8 out of 8	0	Likely Pathogenic (PM1, PM2, PM6, PP3)
KCNQ2* *(NM_172107.2)	067/unknown c.821C>T (p.Thr274Met)	Damaging	Disease-Causing	Probably Damaging	8 out of 8	0	Pathogenic (PS3, PM1, PM2, PP2, PP3, PP5)
	131/de novo c.700A>C (p.Thr234Pro)	Damaging	Disease-Causing	Benign	6 out of 8	0	Likely Pathogenic (PM1, PM2, PM6, PP2)
KMT2B* *(NM_014747.2)	132/de novo c.7551-14_7561del25	NA	NA	NA	NA	0	Pathogenic (PVS1, PM2, PM6)

MBOAT7 (NM_024298.4)	200/paternal c.680_690dup11 (p.Leu231Cysfs*8)	NA	NA	NA	NA	1 in 31356	Likely Pathogenic (PVS1, PM2)
	200/maternal c.758_778del21 (p.Glu253_Ala259del)	NA	NA	NA	NA	5 in 214278	Likely Pathogenic (PM2, PM4, PP1, PP5)
MECP2 (NM_004992.3)	182/de novo c.763C>T (p.Arg255*)	NA	NA	NA	NA	0	Pathogenic (PVS1, PS3, PS4, PM2, PM6, PP5)
NEXMIF (KIAA2022) (NM_001008537.1)	060/unknown c.2163delA (p.Lys721Asnfs*8)	NA	NA	NA	NA	0	Likely Pathogenic (PVS1, PM2)
	158/unknown c.336G>A (p.Trp112*)	NA	NA	NA	NA	0	Likely Pathogenic (PVS1, PM2)
	095/de novo c.1441C>T (p.Arg481*)	NA	NA	NA	NA	0	Pathogenic (PVS1, PM2, PM6, PP5)

PMM2* *(NM_000303.2)	210 and 230/paternal and unknown c.422G>A (p.Arg141His)(1)	Damaging	Disease-Causing	Benign	10 out of 10	891 in 224376	Pathogenic (PS3, PM1, PM5, PP2, PP5)
	210/maternal c.691G>A (p.Val231Met)(1)	Damaging	Disease-Causing	Probably Damaging	10 out of 10	22 in 282644	Pathogenic (PS3, PM1, PM2, PM3, PP2, PP3, PP5)
	230/unknown c.43G>A (p.Gly15Arg)	Damaging	Disease-Causing	Possibly Damaging	9 out of 9	3 in 268534	Likely Pathogenic (PM1, PM2, PM3, PP2, PP3, PP5)
	211/unknown c.710C>T (p.Thr237Met)	Damaging	Disease-Causing	Probably Damaging	10 out of 10	12 in 282470	Pathogenic (PS3, PM1, PM2, PP2, PP3, PP5)
	211/unknown c.447+5G>A (variant IVS5+5G>A)	NA	NA	NA	NA	9 in 169754	VUS (PM2)

	222/unknown c.61C>T (p.Arg21Trp)(1)	Damaging	Disease-Causing	Probably Damaging	9 out of 9	1 in 226464	Likely Pathogenic (PM1, PM2, PP2, PP3)
	222/unknown c.647A>G (p.Asn216Ser)(1)	Damaging	Disease-Causing	Probably Damaging	10 out of 10	7 in 282534	Likely Pathogenic (PM1, PM2, PM5, PP2, PP3, PP5)
	224/unknown c.368G>A (p.Arg123Gln)(1)	Damaging	Disease-Causing	Possibly Damaging	10 out of 10	30 in 223998	Pathogenic (PS3, PM1, PM2, PM3, PP2, PP3, PP5)
	224/unknown c.623G>C (p.Gly208Ala)(1)	Damaging	Disease-Causing	Probably Damaging	10 out of 10	0	Pathogenic (PS3, PM1, PM2, PM3, PP2, PP3, PP5)
PPP2R5D (NM_006245.3)	142/de novo c.592G>A (p.Glu198Lys)	Damaging	Disease-Causing	Probably Damaging	9 out of 9	0	Pathogenic

							(PS3, PM1, PM2, PM6, PP2, PP3)
RNASEH2C (NM_032193.3)	194/maternal c.205C>T (p.Arg69Trp)	Damaging	Disease-Causing	Probably Damaging	5 out of 7	23 in 250836	Pathogenic (PS3, PM2, PP1, PP2, PP3, PP5)
	194/paternal c.348+5G>A	NA	NA	NA	NA	0	VUS (PM2, PM3)
SCN1A (NM_001165963.1)	085/de novo c.635T>C (p.Val212Ala)	Damaging	Disease-Causing	Probably Damaging	6 out of 6	1 in 250484	Likely Pathogenic (PM1, PM2, PM6, PP3)
SCN1A (NM_001202435.1)	157/unknown c.4934G>A (p.Arg1645Gln)	Damaging	Disease-Causing	Probably Damaging	6 out of 6	0	Pathogenic (PS2, PM1, PM2, PM5, PP2, PP3, PP5)
SCN2A (NM_021007.2)	205/paternal (symptomatic) c.2828_2829delGGinsAT	NA	NA	NA	NA	0	VUS (PM1, PM2, PP2)

	(p.Trp943Tyr)						
	202/paternal c.2659G>A (p.Val887Ile)	Damaging	Disease-Causing	Probably Damaging	6 out of 6	0	Likely Pathogenic (PM1, PM2, PP2, PP3)
SLC2A1* *(NM_006516.2)	009/de novo c.940G>A (p.Gly314Ser) (	Damaging	Disease-Causing	Probably Damaging	8 out of 8	0	Pathogenic (PS1, PS3, PM1, PM2, PM6, PP2, PP3, PP5)
	012/de novo c.656delA (p.Asn219Thrfs*10)	NA	NA	NA	NA	0	Pathogenic (PVS1, PM2, PM6)
SLC6A1* *(NM_003042.3)	177/de novo c.881_883delTCT (p.Phe294del)	NA	NA	NA	NA	0	Likely Pathogenic (PS2, PM2, PP5)
SLC35A2* *(NM_001042498.2)	015/de novo c.3G>A (p.Met1?)	Damaging	Disease-Causing	Benign	N/A	0	Pathogenic (PVS1, PM2, PM6, PP5)

***STXBP1* (NM_003165.3)**	001/de novo c.560C>T (p.Pro187Leu)	Damaging	Disease-Causing	Probably Damaging	10 out of 10	0	Likely Pathogenic (PM1, PM2, PM6, PP3)
***SYNGAP1* (NM_006772.2)**	061/de novo c.1490A>G (p.Tyr497Cys)	Damaging	Disease-Causing	Probably Damaging	6 out of 9	0	VUS (PM2, PM6)
***TCF20* (NM_005650.1)**	155/de novo c.3997delC (p.Leu1333Serfs*18)	NA	NA	NA	NA	0	Pathogenic (PVS1, PM2, PM6)
***UBE3A* (NM_130838.1)**	117/de novo c.1396A>G (p.Lys466Glu)	Damaging	Disease-Causing	Probably Damaging	10 out of 10	0	Likely Pathogenic (PM1, PM2, PM6, PP3, PP5)
***WDR45* (NM_007075.3)**	004/de novo c.400C>T (p.Arg134*)	NA	NA	NA	NA	0	Pathogenic (PVS1, PM2, PM6, PP5)

1. Al Teneiji A, Bruun TU, Sidky S, Cordeiro D, Cohn RD, Mendoza-Londono R, Moharir M, Raiman J, Siriwardena K, Kyriakopoulou L, Mercimek-Mahmutoglu S 2017 Phenotypic and genotypic spectrum of congenital disorders of glycosylation type I and type II. Mol Genet Metab 120:235-242.
